# Advancements in Beta-Adrenergic Therapy and Novel Personalised Approach for Portal Hypertension: A Narrative Review

**DOI:** 10.3390/life15081173

**Published:** 2025-07-24

**Authors:** Raluca-Ioana Avram, Horia Octav Minea, Laura Huiban, Ioana-Roxana Damian, Mihaela-Cornelia Muset, Simona Juncu, Cristina Maria Muzica, Sebastian Zenovia, Ana Maria Singeap, Irina Girleanu, Carol Stanciu, Anca Trifan

**Affiliations:** 1 Department of Gastroenterology, Grigore T. Popa University of Medicine and Pharmacy, 700115 Iasi, Romania; ralucaioanaavram@gmail.com (R.-I.A.); ioana.galatanu@gmail.com (I.-R.D.); l_muset@yahoo.com (M.-C.M.); simona.junca@yahoo.com (S.J.); lungu.christina@yahoo.com (C.M.M.); sebastianzenovia20@gmail.com (S.Z.); anamaria.singeap@yahoo.com (A.M.S.); gilda_iri25@yahoo.com (I.G.); stanciucarol@yahoo.com (C.S.); ancatrifan@yahoo.com (A.T.); 2 Institute of Gastroenterology and Hepatology, “St. Spiridon” University Hospital, 700111 Iasi, Romania

**Keywords:** liver cirrhosis, beta-blockers, beta-3 adrenergic receptor, biomarkers

## Abstract

Liver cirrhosis is a chronic progressive disease marked by the transition from a compensated to a decompensated stage, associated with severe complications. Central to this progression is portal hypertension, which results from increased intrahepatic vascular resistance and endothelial dysfunction, as well as splanchnic vasodilation and an augmented circulatory state. Non-selective beta-blockers (NSBBs) remain the standard of care for portal hypertension, reducing portal pressure by lowering cardiac output via beta-1 receptor blockade and decreasing splanchnic blood flow through beta-2 receptor antagonism. However, clinical application of NSBBs is often hindered by adverse effects such as bradycardia, hypotension, and fatigue, alongside inconsistent efficacy in certain patient populations. Such limitations have driven the search for alternative therapeutic strategies and effective biomarkers for identifying non-responders. Beta-3 adrenergic receptor agonists have emerged as promising candidates, acting through distinct mechanisms, different from NSBBs. By stimulating nitric oxide release from endothelial cells, beta-3 agonists induce selective vasodilation without negatively impacting cardiac function, potentially overcoming the limitations of traditional therapies. This review discusses the molecular pathways of NSBBs, their clinical role and limitations, introduces potential novel biomarkers, and highlights the growing evidence supporting beta-3 receptor agonists as novel and targeted treatments for portal hypertension.

## 1. Introduction

Liver cirrhosis represents the terminal phase of progressive chronic liver disease, characterized by advanced fibrosis, architectural disruption, and regenerative nodule formation, posing a significant challenge in public health due to frequent underdiagnosis in the predominantly asymptomatic compensated stage [1]. The disease’s natural course transitions from compensated to decompensated cirrhosis, associated with markedly decreased life expectancy, with mortality risks increased 5-fold and 10-fold, respectively, compared to the general population [2,3]. Reported survival rates differ considerably between compensated and decompensated states, 87% vs. 75% over one year, and 67% vs. 45% over five years [4,5].

The progression towards decompensated cirrhosis is driven by portal hypertension and its associated hyperdynamic circulation, which precipitates key complications such as ascites, gastroesophageal variceal bleeding, and hepatic encephalopathy. A comprehensive understanding of portal hypertension’s underlying mechanics is essential for identifying early diagnostic issues and therapeutic targets to prevent liver decompensation [5].

Decompensation rates range from 4 to 12% annually, based on disease aetiology [5]. Beyond increased mortality, the decompensated stage substantially diminishes quality of life and carries a significant healthcare burden due to heightened hospitalisation rates, treatment demands, and productivity losses. In this context, preventing transition to decompensation and sustaining compensated status is of utmost importance [6].

Recent advancements in our understanding of molecular mechanisms have opened new possibilities for targeted therapies. Beta-adrenergic therapy continues to be a promising strategy for reducing portal hypertension and preventing decompensation. Beta-blockers, such as propranolol and carvedilol, act by decreasing splanchnic blood flow and modulating systemic hemodynamics. Beyond their well-established hemodynamic effects in portal hypertension, non-selective beta-blockers (NSBBs) are increasingly recognized for their antifibrotic potential in liver cirrhosis. Experimental and preclinical studies have demonstrated that adrenergic signalling plays a crucial role in hepatic fibrogenesis by activating hepatic stellate cells (HSCs). Activation of α- and β-adrenergic receptors drives the transition of quiescent HSCs into active myofibroblast-like cells, which produce collagen and other components of the extracellular matrix, contributing to increased intrahepatic vascular resistance and the progression of fibrosis [7,8]. Pharmacological blockade of these pathways using agents such as carvedilol or doxazosin has been associated with decreased HSC activation, downregulation of profibrotic genes (such as α-SMA and Col1a1), and induction of cellular senescence, leading to fibrosis regression in experimental models of cirrhosis induced by carbon tetrachloride (CCl_4_) or bile duct ligation [7]. Furthermore, nanotechnology-based strategies have recently been explored to enhance the specificity and efficacy of adrenergic therapy in fibrotic liver tissue. A study published by Fei et al. demonstrated that a biomimetic nanoplatform composed of retinoic acid and labetalol successfully targeted activated HSCs, resulting in a significant reduction in hepatic inflammation, lipid accumulation, and collagen deposition [8]. The results support the hypothesis that adrenergic blockade may exert direct antifibrotic effects, offering a complementary therapeutic strategy for the management of cirrhosis, especially in its compensated stages. Furthermore, beta-adrenergic blockade is involved in restoring immune homeostasis by attenuating stress-induced immunosuppression, thus potentially reducing the risk of infections—a key driver of decompensation [9].

Despite potential benefits, challenges persist in optimising beta-adrenergic therapy for compensated cirrhosis. Heterogeneity in patient responses, driven by genetic polymorphisms in adrenergic receptor pathways and variations in microbiota-derived metabolites, underscores the need for personalised approaches [7,10]. Additionally, the adverse effects associated with current beta-blockers, such as fatigue and bradycardia, limit their broader application. A deeper understanding of the molecular underpinnings of portal hypertension and fibrosis is essential to refine therapeutic strategies [10].

This narrative review aims to explore the recent advancements in beta-adrenergic therapies for compensated liver cirrhosis by synthesizing current evidence and highlighting emerging molecular insights.

## 2. Materials and Methods

A comprehensive literature search was conducted using PubMed, Scopus, and Web of Science to identify peer-reviewed articles related to liver cirrhosis, beta-blockers, beta-3 adrenergic receptor, and biomarkers. The search included studies published between 2010 and 2024, with a focus on the most recent five years (2020–2024), reflecting the growing interest in beta-adrenergic pathways.

Keyword combinations such as “liver cirrhosis”, “beta-blockers”, “beta-3 adrenergic receptor”, and “biomarkers” were used both as free-text terms and MeSH entries. Reference lists of relevant publications were also screened to identify additional eligible studies.

Articles were included if they were peer-reviewed, published in English, and addressed hepatic fibrogenesis, pathophysiology, or therapeutic interventions targeting beta-adrenergic mechanisms. Original research articles and clinical case reports were eligible, while editorials, expert opinions without primary data, and preprints were excluded.

Out of approximately 1500 records initially retrieved, 91 studies were selected for inclusion. More than 60% were published between 2020 and 2024. Among them, 53 were original articles—including 14 experimental studies, 11 clinical studies, 5 randomized controlled trials, 9 mechanistic investigations, and 14 observational studies. An additional 38 review articles and meta-analyses were included based on their relevant conceptual contribution and translational relevance to hepatic fibrosis.

Study inclusion was based on clarity of methodology, scientific relevance, and contribution to understanding adrenergic modulation and biomarker development in cirrhosis. The findings were synthesized thematically to integrate experimental and clinical perspectives. No laboratory experiments, specialized equipment, chemical substances, or dedicated software were used in this literature review.

## 3. Results and Discussions

### 3.1. Portal Hypertension: Pathophysiology and Possible Molecular Targets

**Figure 1:**
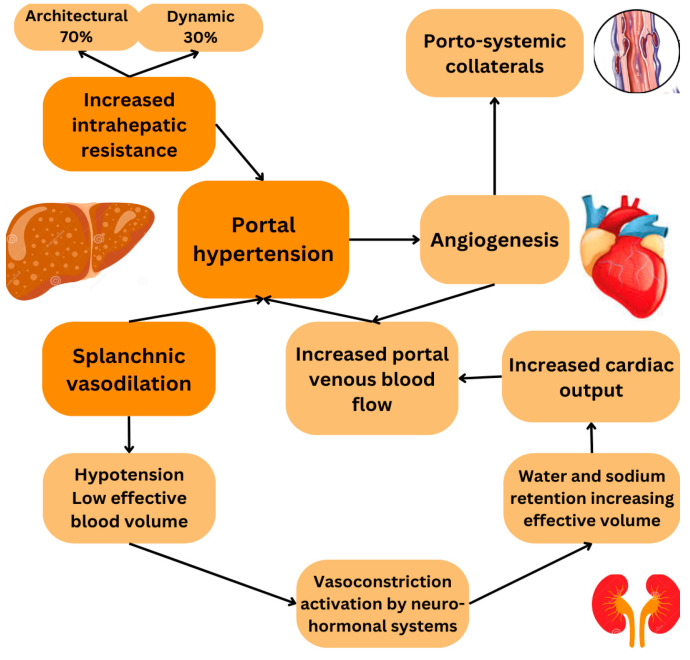
Pathophysiologic mechanisms and hemodynamic consequences of portal hypertension.

Portal hypertension is caused by increased portal pressure and is attributed to both structural obstructions determined by the activation of HSCs and altered extracellular matrix (ECM) remodelling (accounting for 70% of hepatic resistance) and dynamic/humoral factors (30%), such as reduced intrahepatic nitric oxide (NO) levels causing local endothelial dysfunction [9,11,12]. It translates clinically to an increased cardiac output and expanded plasma volume in the splanchnic vessels, which in the end manifests itself in the form of reduced effective arterial blood volume. Therefore, to counterbalance this effect, hormonal and neurogenic systems, such as the renin–angiotensin–aldosterone and sympathetic systems, are activated. By promoting vasoconstriction, increasing the cardiac output, and increasing water and sodium retention, they attempt to restore the normal effective arterial blood volume [12,13] (Figure 1).

On a molecular level, the upregulation of fibrogenic cytokines, such as transforming growth factor-beta (TGF-β) and platelet-derived growth factor (PDGF), promotes the differentiation of quiescent HSCs into myofibroblast-like cells, exacerbating fibrosis and portal pressure. Furthermore, the dysregulation of NO and endothelin-1 within the intrahepatic vasculature contributes to increased vascular tone, perpetuating portal hypertension [11]. These molecular pathways not only fuel fibrosis but also set the stage for complications such as gastroesophageal variceal bleeding, ascites, and spontaneous bacterial peritonitis.

The hyperdynamic circulatory state associated with portal hypertension is established by the splanchnic vasodilation caused by an increased production of vasodilators, along with a reduced contractile response induced by shear stress, inflammatory cytokines, and vascular growth factors, such as vascular endothelial growth factor (VEGF) that stimulate angiogenesis, leading to collateral vessel formation [12,14]. Once collateral and arterial vasodilation develop in splanchnic and systemic circulations, portal flow increases, resulting in reduced effective arterial blood volume, which in turn results in the compensatory activation of the neurohormonal systems [12,15].

### 3.2. The Mechanisms of Classic Beta-Adrenergic Receptors in Cirrhosis: Beta-1 and Beta-2

In decompensated cirrhosis, patients exhibit elevated plasma norepinephrine (NE) levels, reflecting sympathetic overactivation driven by arterial hypovolemia and baroreceptor stimulation [9]. The increased NE concentrations correlate with elevated vasopressin, renin, and aldosterone levels, exacerbating vasoconstriction and fluid retention. This amplified adrenergic activity contributes to the progression of portal hypertension and renal dysfunction and closes the loop. The dysregulated adrenergic system is characterised by localised increases in NE production within the kidney, liver, and heart, even when systemic plasma levels do not fully reflect this activity [8,12,13,15,16,17,18].

Catecholamines (norepinephrine and epinephrine) exert their effects through both β-adrenergic (β1, β2, and β3) and α-adrenergic (α1 and α2) receptors, all of which are coupled to G proteins. The distribution of these receptors in various organs dictates their physiological and hemodynamic impact [9,18].

Beta-blockers exert their therapeutic effects by targeting these adrenergic receptors and modulating portal pressure and splanchnic hyperperfusion. Their interaction with β-adrenergic receptors generates distinct hemodynamic and antifibrotic responses, which are critical in managing portal hypertension and slowing disease progression [9,12,19,20,21,22,23].

- Beta-1 Adrenergic Mechanism

The beta-1 adrenergic receptor (β1AR) is a G-protein-coupled receptor that signals through the Gs alpha subunit. Activation of this pathway stimulates adenylyl cyclase, leading to the conversion of adenosine triphosphate (ATP) into cyclic adenosine monophosphate (cAMP) and initiating a cAMP-dependent signalling cascade. The increased levels of cAMP activate protein kinase A (PKA), which phosphorylates calcium channels, thereby enhancing cellular calcium influx and amplifying the functional response of the receptor [20,21]. Blocking β1 receptors, predominantly found in the myocardium, leads to a reduction in heart rate and cardiac output, thereby decreasing splanchnic blood flow and ultimately lowering portal pressure. By suppressing sympathetic activity and inhibiting the renin–angiotensin–aldosterone system, β1 blockade mitigates compensatory vasodilation and the hyperdynamic circulation characteristic of cirrhosis [22,23].

b. Beta-2 Adrenergic Mechanism

The activation of β2-adrenergic receptors (β2AR) in cirrhosis triggers a Gs protein-mediated signalling cascade, leading to increased cAMP production and activation of PKA [23,24]. PKA phosphorylates and activates endothelial nitric oxide synthase (eNOS), elevating NO levels and promoting systemic and splanchnic vasodilation, a key factor in reduced vascular resistance and portal hypertension. Inhibiting β2 receptors on splanchnic vessels induces controlled vasoconstriction, reducing splanchnic blood flow and alleviating the excessive perfusion of the portal system. Additionally, β2 blockade limits the release of endogenous vasodilators, including prostaglandins and NO, thereby counteracting pathological vasodilation and restoring hemodynamic balance in cirrhotic patients [9,24,25].

### 3.3. Non-Selective Beta Blockers: Traditional and Carvedilol

Carvedilol, a third-generation NSBB, enhances these effects through additional alpha-1 receptor blockade, leading to intrahepatic vasodilation and achieving a greater reduction in portal pressure compared to traditional NSBBs [26]. The dose-dependent effects of carvedilol may also explain its better tolerance in patients, as moderate decreases in heart rate and cardiac output reduce adverse cardiovascular impacts [22]. At doses exceeding 25 mg/day, carvedilol reduces mean arterial pressure (MAP), an effect not observed at lower doses (6.25–25 mg/day), where it demonstrates superior efficacy in reducing portal hypertension compared to nadolol or propranolol [21,22,23,24,25,27,28,29,30,31]. Zacharias et al. [25] confirm carvedilol’s superiority over traditional beta-blockers in lowering HVPG by analysing six clinical trials with no statistical heterogeneity (I^2^ = 0%), with a pool of 368 participants. Their systematic review showed that carvedilol lowered the HVPG by 8% more than other NSBBs, with a CI of 95%. Moreover, no significant differences were noted in rates of complications, variceal bleeding, or mortality, with an RR of 0.77 and 95% CI of 0.43–1.37 for upper gastrointestinal bleeding and an RR of 0.97 and 95% CI of 0.67–1.42 for serious adverse effects [25]. Villanueva et al. further demonstrated through a time-to-event meta-analysis that carvedilol therapy significantly improves survival and reduces the risk of decompensation, especially in cases of ascites, thereby solidifying carvedilol as the preferred first-line therapy for managing portal hypertension. Their study proved that carvedilol lowered the risk of developing decompensated cirrhosis by approximately 50% (HR = 0.506), with a 95% CI = 0.289–0.887, *p* = 0.017 in comparison to the control group. This protective effect appeared to be primarily driven by the reduction in the risk of ascites development, which was achieved with an HR of 0.491, 95% CI 0.247–0.974, *p* = 0.042. Additionally, carvedilol lowered the risk of death by approximately 58%, with an HR of 0.417, 95% CI = 0.194–0.896, *p* = 0.025 [27]. The studies that prove the superiority of carvedilol in compensated cirrhosis are shown in Table 1.

Carvedilol, unlike most other NSBBs, acts as a *biased ligand*, not only blocking β-adrenergic receptors but also activating β-arrestin-induced signalling pathways [9]. β-arrestins (β-arrestin-1 and β-arrestin-2) are intracellular proteins that regulate G-protein-coupled receptor (GPCR) signalling [20,34,35,36]. They traditionally function by desensitising G-protein signalling via receptor endocytosis but also act as scaffold proteins, forming intracellular signalosomes that activate additional pathways [35,36]. This behaviour is distinct from classical GPCR signalling and is linked to mitogen-activated protein kinase (MAPK) activation, potentially affecting cell proliferation, differentiation, and growth [9,20,36]. In liver cirrhosis, an increased expression of β-arrestin-2 has been documented in the liver, gastric mucosa, and splanchnic vessels, suggesting that β-arrestin overexpression may be a factor for impaired vascular responses to vasoconstriction [37,38]. On the other hand, a deficiency of β-arrestin-2 in sinusoidal endothelial cells (SEC) has been implicated in the reduced NO production and increased intrahepatic vascular resistance characteristic of cirrhosis [39].

Carvedilol’s biased signalling through β-arrestin pathways distinguishes it from traditional NSBBs like propranolol [9]. The presumed effects of β-arrestin activation in cirrhosis include reduced splanchnic vasodilation, lowering portal tributary blood flow, and portal pressure. It is hypothesised that carvedilol’s effects are amplified due to β-arrestin upregulation in splanchnic vessels, potentially enhancing its efficacy in portal pressure reduction compared to traditional NSBBs [9,35,36,37]. In a prospective study by Lashen et al., it was suggested that β-arrestin serum levels and expression in gastric antrum mucosa may serve as biomarkers for predicting hemodynamic responses to acute NSBB administration. Patients who exhibit higher β-arrestin levels have been found to respond better to NSBB therapy, 95.1% of the patients with high expression of β-arrestin levels being responders to NSBB therapy (*p* < 0.001), while of those with low levels of β-arrestin, only 17.6% were NSBB responders (*p* < 0.001), although the interpretation of these findings is limited by the lack of cell-specific data. Additionally, they reported that a higher expression of β-arrestin-2 was associated with a longer variceal bleeding-free interval under NSBBs. In their study, strong β-arrestin-2 expression was found in 41 patients, from which only 2 experienced variceal bleeding (*p* < 0.001) [9,35,36]. While the exact functional outcomes of β-arrestin signalling in cirrhosis, particularly its effects on portal hypertension, splanchnic vascular cells, and cardiac function, remain unclear, its upregulation in liver disease suggests a significant role in disease progression [35].

The role of NSBBs in HCC (hepatocellular carcinoma) development remains to be debated [6,13,39,40]. Cheng et al., in his longitudinal study that included patients with chronic B hepatitis without liver cirrhosis determined that no significant benefit of HCC risk reduction was observed between the NSBB users and nonusers (adjusted HR, 0.82; 95% CI, 0.52–1.31) [40], while Chang et al. showed that there might be an improvement in overall survival in unresectable or metastatic HCC for patients under propranolol. Their statistical analysis showed a 22% reduction in mortality risk associated with propranolol administration, with an HR = 0.78 (95%CI = 0.72–0.84, *p* < 0.001) [41].

Additionally, the window of opportunity for using NSBBs in complicated liver cirrhosis is narrow. The decision to administer NSBBs in cases of refractory ascites, especially when associated with severe arterial hypotension, active infection, or acute kidney injury, should be taken carefully, and at the very least, a dose reduction should be considered. In most cases, acute events represent a contraindication for NSBBs, because they cannot achieve a fast and significant portal venous decompression [14].

Regarding the role of NSBBs in liver transplantation, despite the proven superiority of carvedilol over propranolol in lowering portal pressure (Table 1), carvedilol’s stronger effect on lowering arterial blood pressure can increase the need for diuretics and may offer less survival benefit compared to propranolol in cirrhotic patients with ascites who are on the transplant list. Therefore, while carvedilol is generally preferred for primary prophylaxis in compensated cirrhosis, its use is avoided in transplant candidates with severe or refractory ascites or those experiencing progressive arterial hypotension. This tailored approach optimises patient stability and outcomes while waiting for transplantation [42].

### 3.4. Personalised NSBB Therapy in Cirrhosis: Integrating Biomarkers, Genetics, and Non-Invasive Monitoring

- Biomarkers of Non-Invasive Monitoring of NSBB Efficacy in Cirrhosis

The variable hemodynamic efficacy of NSBBs requires individualised monitoring, which has traditionally relied on invasive procedures such as HVPG measurements. The search for reliable non-invasive markers has led to the exploration of soluble vascular cell adhesion molecule-1 (sVCAM-1) as a potential indicator of the hemodynamic response to NSBBs [43].

Santos et al., in the preliminary analysis of their ongoing double-blind and randomised clinical trial, demonstrated that serum sVCAM-1 levels were significantly higher in patients with decompensated cirrhosis, reflecting increased shear stress and systemic inflammation contributing to endothelial dysfunction (*p* < 0.01). Furthermore, a moderate correlation between HVPG variation and sVCAM-1 levels after NSBBs treatment suggests that sVCAM-1 may reflect changes in portal pressure and hepatic vascular resistance (*p* = 0.03). These findings highlight its potential as a non-invasive marker for monitoring NSBB efficacy, although further studies are needed to validate its clinical application and establish thresholds for therapeutic decision-making [43].

Serum ferritin (FTH) triggers hepatic inflammation by activating the NOD-like receptor protein 3 (NLRP3) inflammasome through intercellular adhesion molecule-1 (ICAM-1) on HSCs, resulting in increased interleukin-1 beta (IL-1β) production. This pathway, driven by clathrin-mediated endocytosis and lysosomal destabilisation, contributes to hepatic fibrogenesis in chronic liver disease [44]. While NSBBs are known to modulate inflammatory responses in cirrhosis [44,45,46,47], direct evidence linking NSBB therapy to reduced ICAM-1 expression is currently lacking [48]. However, ICAM-1 expression is induced by inflammatory cytokines such as Tumor Necrosis Factor alpha (TNF-α) and IL-1β through the activation of the nuclear factor kappa B (NF-κB) pathway [44,47,48,49]. Since NSBBs could attenuate systemic inflammation, it is plausible that they may indirectly influence ICAM-1 expression. Further research is needed to elucidate the specific effects of NSBBs on ICAM-1 levels in cirrhotic patients [17,47,48,49,50,51].

NSBBs contribute to reducing systemic and liver inflammation in cirrhosis through adrenergic blockade and the reduction in norepinephrine, a key mediator of endothelial dysfunction and exaggerated inflammatory responses [18,43,51]. Elevated levels of interleukin-6 (IL-6), a central inflammatory factor correlated with disease severity (Child–Pugh, Model for End-Stage Liver Disease—MELD, Chronic Liver Failure–Sequential Organ Failure Assessment—CLIF-SOFA), are reduced by NSBBs through decreased intestinal permeability, reduced bacterial translocation, and direct effects on IL-6-producing cells [18,44,45,46,47,48,49]. Almenara et al. showed in their prospective longitudinal study that higher levels of IL-6, interleukin-10 (IL-10), and interferon-gamma (IFN-γ) were identified in patients who developed more clinical events, and the ones treated with NSBBs had a significantly better event-free survival, including HCC, death, and the need for liver transplantation in a multivariate Cox regression adjusted for Child–Pugh score, esophageal varices, and platelets (HR: 0.36, 95% CI: 0.18–0.71) [45]. The chronic immunomodulatory effect of NSBBs on these inflammatory factors partly explains their ability to reduce complications and slow disease progression. Additionally, NSBB therapy was shown to reduce levels of IL-6, intestinal permeability, and bacterial translocation, contributing to lower intrahepatic resistance and improved outcomes [46,47]. In a study by Reiberger et al., the HVPG was measured before and during treatment with NSBBs, together with the intestinal permeability, and an amelioration of gastroduodenal/intestinal permeability and a decrease in bacterial translocation in patients under NSBBs treatment (LBP—16% *p* = 0.018; IL-6–41% *p* < 0.0001) was discovered [46].

Studies highlight the roles of endothelin-1 (ET-1), transforming growth factor-beta (TGF-β), and von Willebrand factor (vWF) as key mediators in the progression of portal hypertension and their potential use as non-invasive biomarkers for assessing disease severity and monitoring therapeutic response in cirrhosis [52,53,54,55].

In a prospective, observational study by Wereszczynka-Siemiatkowska et al., ET-1 and TGF-β2 peripheral levels were measured before and after NSBB therapy, showing that peripheral ET-1 levels significantly increased (*p* = 0.032), while the peripheral levels of TGF-β2 significantly decreased and maintained their low levels, compared to the controls (*p* = 0.002). Also, the study showed that in patients with normalised HVPG under NSBB therapy, defined as (HVPG < 5 mmHg), had lower levels of peripheral TGF-β2 in comparison to patients without HVPG normalisation (*p* = 0.042) [53].

Additionally, vWF, a marker of endothelial dysfunction, was proposed as a predictor of portal hypertension due to its role in vascular remodelling and activation [54,55]. In a retrospective study by Pomej et al., increased levels of vWF were significantly correlated with disease severity (increased levels of vWF were identified in 4% of the patients with compensated advanced chronic liver disease (cACLD) stage 0, in 10% of the patients with cACLD 1 and in 23% of the patients with cACLD 2–4, with *p* < 0.011) and were predictive of decompensation/liver-related mortality, for each increase of 10% for the vWF levels, being an associated 2% increase in risk of decompensation or mortality (HR = 1.02 with a 95%CI = 1.01–1.04, *p* < 0.08 [55].

A promising area of research involves circulating biomarkers such as Micro-RNA-181 b-5p (*miR-181b-5p*), which has been identified as a potential marker for predicting systemic circulatory dysfunction in decompensated cirrhosis, though its clinical utility requires further validation [56,57]. Garcia Garica de Paredes et al. demonstrated in their prospective study that miR-181b-5p levels at 1 year, but not at baseline, for patients who achieved HVPG normalization were higher in patients that developed ascites, in comparison to the ones that did not, thus being a possible non-invasive marker efficient in predicting decompensation (ascites) (the AUC of miR-181b-5p at 1 year to predict ascites was 0.7 (95% CI 0.59–0.78)) [57].

By enabling non-invasive monitoring of therapeutic response and progression of portal hypertension, these biomarkers could help refine NSBB treatment regimens more effectively. However, further large-scale studies are needed to validate their clinical significance and optimise their use in personalised treatment strategies [18,56,57]. The main findings associated with the novel biomarkers are illustrated in Table 2.

b. Genetic Polymorphism in Beta-Blocker Response

Inter-patient variability in response to NSBBs is partly driven by genetic polymorphisms, particularly in genes related to β-adrenergic signalling and drug metabolism [18,65,66]. The most clinically relevant genes affecting NSBB response include Cytochrome P450 2D6 (CYP2D6), β1-adrenergic receptors (ADRB1), beta-2-adrenergic receptor (ADRB2), and G protein-coupled receptor kinases 5 (GRK5) [67]. Among these, ADRB2 polymorphisms have the greatest clinical impact, as they directly impair vascular responses and reduce receptor sensitivity to catecholamines, leading to non-responsiveness. In contrast, CYP2D6 polymorphisms primarily affect drug metabolism, but their overall clinical impact on NSBB therapy remains limited [65,66].

Polymorphisms in the ADRB2 alter receptor affinity for catecholamines [65,66,67,68], leading to inadequate β-adrenergic blockade [67] and explaining why 30–60% of patients are classified as non-responders to NSBB therapy [10,68,69]. These variations impair vascular responses and reduce the effectiveness of propranolol and carvedilol in lowering portal pressure, making ADRB2 polymorphisms a critical factor in NSBB efficacy [68]. A study by Kong et al. showed that patients exhibiting a specific polymorphism of the ADRB2 gene—Gly16-Glu/Gln27 homozygotes, achieved lower variceal pressures in response to propranolol administration (a decrease of 22.4% ± 2.1%) compared to Arg16Gln27 homozygotes (a decrease of 13.1% ± 2.7%) (*p* < 0.01) [65].

Similarly, variations in the CYP2D6 gene, responsible for propranolol metabolism, influence plasma drug concentrations. Intermediate and poor metabolizers exhibit higher plasma drug levels at standard doses, potentially leading to effective β-blockade at lower doses [65,66,67,68]. However, the CYP2D6 genotype generally has minimal impact on adverse effects or overall outcomes [66,67]. A study by Zhang et al. evaluated the relationship between the hemodynamic response to propranolol and CYP2D6 and ADRB2 gene polymorphisms in Chinese Han patients and indicated that the CYP2D6 (188C > T) genotype could be an independent predicting factor for HVPG response to propranolol (*p* =  0.033) [66].

These data suggest that integrating pharmacogenetic data from both ADRB2 and CYP2D6 could enhance precision-based NSBB therapy. Early identification of non-responders through genetic testing could enable timely dose adjustments or the selection of alternative therapies, ultimately improving therapeutic outcomes and reducing complications such as portal hypertension, spontaneous bacterial peritonitis (SBP), and HCC [18,65,66]. Additionally, there is a high number of clinical outcomes studies and consistent data in favour of testing the ADRB1 genotype, similar to many other genetic tests that are already in clinical use and approved by the Clinical Pharmacogenetics Implementation Consortium Guidelines [67]. Such an example is the INVEST trial, in which Ser49-Arg389 haplotype of the ADRB1 genotype was associated with increased risk of death by a major adverse cardiovascular event (HR = 3.66, 95% CI: 1.68–7.99) and after administering a betablocker (Atenolol), the risk of death was significantly reduced (HR = 2.31, 95% CI: 0.82–6.55; *p* = 0.11) [67].

Although CYP2D6 is currently the only gene used in clinical practice [66], it is not routinely applied to guide beta-blocker therapy. Current evidence does not support ordering a CYP2D6 pharmacogenetic test specifically for NSBB management. The limitations of the study are represented by a relatively low number and homogeneous group of patients included, primarily from a single centre and of a similar ethnicity; thus, the results may not be universally applicable and require a more diverse population study. To implement genotype testing, additional resources are required, alongside laboratory facilities and trained personnel, which may not be justifiable in all healthcare settings due to the high costs involved, limited resources, and the low price of propranolol, which is already widely used in clinical practice based on already existing guidelines [67,68]. However, when CYP2D6 genotype data are available, they may help clinicians adjust doses to achieve the target heart rate response. Given the importance of ADRB2 and other receptor-related polymorphisms, future research should focus on developing integrated pharmacogenetic panels that combine receptor sensitivity data (ADRB2) and drug metabolism profiles (CYP2D6) to tailor NSBB therapy. Further integration of pharmacogenetics into precision medicine is essential to optimise NSBB therapy, improve response rates, and minimise the risk of complications related to portal hypertension and disease [65,66,67,68].

### 3.5. Beta-3 Adrenergic Mechanism—Emerging Roles in Liver Disease

Following the initial identification of β1 and β2 adrenoceptors, it was discovered that certain β-adrenergic responses do not align with either subtype, leading to the eventual cloning of the β3-adrenergic receptor (β3-AR) in 1989 [70,71]. Since then, β3-AR has been identified in several tissues, revealing its involvement in various signalling pathways and physiological functions, as summarised in Table 3.

**Figure 2:**
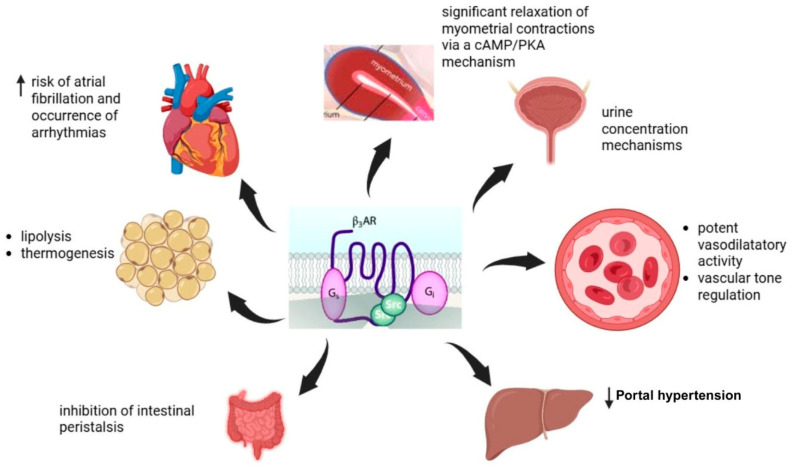
β3-adrenergic receptors: tissue localisation and functional outcomes.

β3AR is involved in various physiological processes across different organ systems. Firstly, in adipose tissue, it promotes lipolysis and thermogenesis, and in the cardiovascular system, it is associated with an increased risk of arrhythmias such as atrial fibrillation. Additionally, β3AR stimulation inhibits intestinal peristalsis in the gastrointestinal tract and induces a cAMP/PKA-mediated relaxation of contractions in the myometrium. Moreover, in the urinary system, it supports urine concentration mechanisms. Furthermore, β3AR stimulation may play a crucial role in regulating vascular tone through vasodilation. Activation of β3AR in the liver has also been linked to reduced portal pressure. The expression of β3AR activation is illustrated in Figure 2.

However, its functional role remains controversial and only partially understood. Recent research highlights its role in urine concentration mechanisms, fat mass reduction, and inflammatory processes, suggesting novel therapeutic applications [62,63,64,65,66,67]. Additionally, stimulation of β3-AR is associated with potent vasodilatory activity and plays a role in vascular tone regulation [65,68]. This property positions β3 receptors as a promising therapeutic target, offering an alternative pharmacological approach for reducing portal pressure in non-responders or in patients experiencing adverse reactions to conventional beta-blockers [24,25,27]. Moreover, it plays a crucial role in lipolysis and thermogenesis in brown adipose tissue. Research suggests that sympathetic nerve activation and abnormal β3-AR signal transduction play a significant role in the onset and development of metabolic syndrome, including obesity, hypertension, diabetes, and hyperlipidaemia [70,71,78].

Activation of β3 receptors stimulates NO release from endothelial cells, promoting selective vasodilation of splanchnic vessels and reducing portal pressure without negatively impacting cardiac output [73,74]. Moreover, β3 receptor stimulation can ameliorate endothelial dysfunction, inhibit HSC activation, and reduce fibrosis progression through enhanced vasodilatory and antifibrotic effects. It is observed that β2- and β3-adrenergic receptors are involved in NO production, with β2AR playing a dominant role in acute vasodilation and liver regeneration, while β3AR is more associated with metabolic regulation and NO-mediated vasodilation in chronic or metabolic contexts, such as insulin resistance and endothelial dysfunction [73,75,76,79,80].

- Beta 3 effects in fat metabolism

Adipose thermogenesis is a key therapeutic target for addressing metabolic dysfunction in obesity. However, its effectiveness in middle-aged and elderly individuals, who have the highest obesity prevalence, remains uncertain due to age-related declines in thermogenesis. Animal studies have shown that chronic stimulation with the β3-adrenergic agonist CL316,243 (CL) reduces fat mass, increases energy expenditure, fatty acid oxidation, mitochondrial activity, and improves glucose homeostasis and the adipokine profile [70,81].

At the cellular level, β3-AR treatment activates uncoupling protein 1 (UCP1)-dependent thermogenesis in brown adipose tissue (BAT) and enhances lipolysis and de novo lipogenesis (DNL) in white adipose tissue (WAT), triggering the futile lipid cycle independently of UCP1. These findings highlight that chronic β3-AR stimulation activates distinct metabolic pathways in BAT and WAT, promoting energy expenditure and systemic metabolic improvements in aged animals. Given the loss of BAT with ageing, activating the futile lipid cycle in WAT could serve as a novel strategy to combat age-related metabolic dysfunctions [70,81].

Furthermore, metabolic associated steatotic liver disease (MASLD), closely linked to adipose dysfunction, insulin resistance, and chronic inflammation, may benefit from β3-AR stimulation. By reducing ectopic fat accumulation, enhancing fatty acid oxidation, and improving metabolic flexibility, this approach could help mitigate hepatic steatosis and inflammation, offering a complementary strategy for managing liver metabolic disease and, furthermore, for the prevention of cirrhosis [70,81,82,83].

Experimental studies involving both stimulation and inhibition of β3-AR expression have shown that long-term stimulation of β3-AR can alleviate hepatic lipid accumulation, hepatic steatosis, and inflammation associated with MASLD. These effects are likely linked to the regulation of peroxisome proliferator-activated receptors (PPAR)/carnitine palmitoyl transferase I (CPT-1) and fatty acid translocase (FAT/CD36) expression [75,79]. Activation of β3-AR has been shown to alleviate lipid accumulation in hepatocytes within the context of MASLD. This effect is linked to its regulation of key proteins involved in fatty acid (FA) metabolism [41,42,43,44]. In the livers of rats fed a high-fat diet, stimulation of β3-AR by the agonist BRL37344 upregulated PPAR-α, while β3-AR inhibition had the opposite effect [80]. Therefore, it is proposed that β3-AR activation may interfere with the initiation mechanisms of MASLD [75,77]. Elbadr et al. studied the effects of mirabegron (β3-AR agonist) and silymarin administered orally in obese rats and obese rats with hepatotoxicity induced by carbon tetrachloride (CCl-4). Their results showed a significant reduction in liver enzymes such as alanine transaminase (ALT), aspartate aminotransferase (AST), alkaline phosphatase (ALP), and total bilirubin in the obese group in comparison to the CCl4-treated group (*p* < 0.05) and observed that the obese rats treated with mirabegron and silymarin showed mild histopathological improvements in comparison to the obese rats treated with CCl4 [67]. Wang et al. in his experiment used rats fed with a high-fat diet and after 4 weeks of treatment with a β3-AR agonist (BRL37344), there was a significant reduction in serum liver enzymes (AST, ALT, ALP) and also in the cholesterol panel (triglycerides, total cholesterol, low-density lipoprotein cholesterol—LDL-cholesterol) [80]. These findings indicate that the upregulation of β3-AR expression constitutes a protective mechanism against MASLD, suggesting that β3-AR could be a novel therapeutic target for this condition. Moreover, hepatocellular steatosis, inflammation, and oxidative stress are interdependent processes [77]. Thus, it is speculated that β3-AR activation is a complex event with protective effects against MASLD. Future studies will focus on elucidating the relationship between β3-AR, hepatic inflammation, and oxidative stress in the context of MASLD [77,84].

b. Beta 3 in cirrhosis and portal hypertension

It has been demonstrated that in the case of MASLD, there is an early increase in portal pressure, without significant fibrosis associated, due to sinusoidal compression and reduced vascular compliance [12,59,85]. Thus, the shear stress of blood flow induced by fatty metabolism changes in the liver leads to sinusoidal endothelial dysfunction that is characterized by low local levels of vasodilators such as NO and high synthesis of vasoconstrictors such as ET-1, resulting in increased intrahepatic vascular resistance. Thus, lower NO levels increase portal pressure by promoting vascular resistance and promoting further steatosis by reducing fatty acid β-oxidation, which increases portal pressure, thus closing the loop [32,58,60]. Considering the actions of β3-agonists, stimulation of β3-AR may offer therapeutic benefits in managing portal hypertension through different effects, such as a reduction in inflammation and oxidative stress, resulting from the reduction in hepatic lipid deposits since lipid accumulation is closely linked to subclinical chronic inflammation, a pro-inflammatory state characterized by elevated levels of pro-inflammatory cytokines and increased oxidative stress, which drive fibrogenesis and hepatic tissue stiffening [60,61,62]. Another beneficial effect is the enhancement of endothelial function and reduction in hepatic vascular resistance, both key contributors to portal hypertension, by diminishing hepatic fat accumulation, which restores endothelial function, thereby lowering vascular resistance to blood flow [86]. Additionally, cirrhosis progression can be prevented through limiting structural alterations within the liver induced by lipid accumulation, changes that play a central role in the pathogenesis of portal hypertension [43] and, from the point of view of the metabolic changes associated with cirrhosis, activation of β3 receptors and the subsequent reduction in lipid deposits may ameliorate insulin resistance, acknowledging the established link between impaired insulin sensitivity and the exacerbation of hepatic dysfunction [73,81].

Given its involvement in metabolic regulation, fat metabolism, and inflammatory modulation, β3-AR has gained attention for its potential applications in cirrhosis. In cirrhosis, the altered metabolic environment, systemic inflammation, and hemodynamic dysfunction contribute to disease progression, making β3-AR a potential therapeutic target [78,79]. Notably, the receptor’s common genetic variant Trp64Arg has been associated with metabolic parameters, including cholesterol and glucose tolerance [87]. Activation of β3-AR in adipose tissue can enhance lipolysis and fat mass reduction, potentially alleviating metabolic derangements such as insulin resistance, which are common in cirrhotic patients [79].

**Figure 3:**
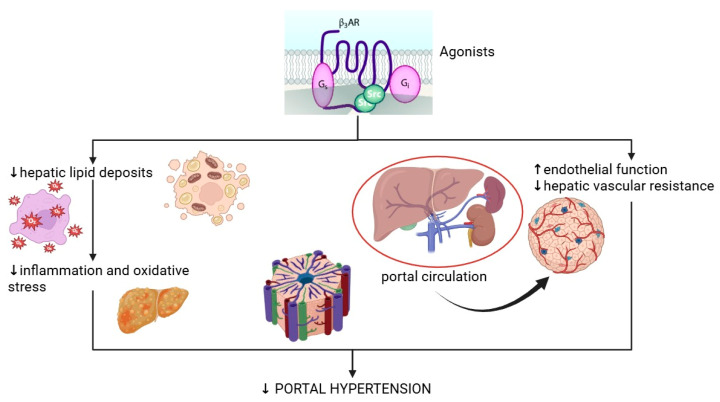
Hepatic and vascular effects of β3 agonism in portal hypertension.

Furthermore, β3-AR-mediated modulation of inflammatory responses could be beneficial in reducing systemic and hepatic inflammation, mitigating complications such as portal hypertension and liver fibrosis. NO regulates inflammation, vasodilation, and oxidative stress by scavenging reactive oxygen species (ROS). It is produced by eNOS, located on endothelial cell membranes alongside β3AR, L-type calcium channels, and caveolin proteins [86,88]. When the β3AR receptor is stimulated by an agonist, eNOS dissociates from caveolin, and the influx of intracellular calcium from the activation of the neighbouring L-type calcium channel activates it, acting also on eNOS itself [32,76,77]. Thus, β3AR stimulation combats oxidative stress directly by targeting nitric oxide production from eNOS and consequently reducing concentrations of ROS. Typically, eNOS is found in uncoupled form in oxidative stress and worsens redox conditions by producing superoxide. After β3AR agonist stimulation, eNOS is recoupled, and redox conditions are improved by stopping superoxide production, increasing production of NO, which in turn diminishes the levels of ROS [76]. Furthermore, the resulting NO from eNOS can promote vasodilation by binding soluble guanylyl cyclase (sGC) and producing cyclic guanosine monophosphate (cGMP) in vascular smooth muscle cells [76,81]. By influencing NO production and vascular tone, β3-AR activation may also help regulate splanchnic vasodilation, a hallmark of cirrhosis-related hemodynamic changes. Agonist binding of the β3AR promotes eNOS recoupling and activation, enhancing NO synthesis and inducing vasodilation [76,77]. This mechanism is crucial for restoring endothelial function under conditions of hyperglycemia and oxidative stress, both of which are common in liver diseases such as MASLD and cirrhosis [76,77]. The effects of β3-AR agonists are summarised in Figure 3.

Vasina et al. demonstrated that stimulation of β3-AR by the selective agonist SR58611A significantly reduced portal pressure (PP) in a dose-dependent manner in cirrhotic rats, obtaining a reduction of approximately 50% of the baseline level with the highest dosage (*p* < 0.05). Furthermore, central venous pressure (CVP) was not significantly affected (−1.4 ± 0.9 mmHg vs. 1.9 ± 0.1 mmHg; *p* < 0.05), indicating that the PP-CVP gradient was reduced, a finding of paramount importance. In fact, it is the increase in this gradient, rather than the portal pressure itself, that is associated with complications related to portal hypertension [73]. These findings suggest that β3-AR may be involved in the pathogenesis of portal hypertension and could represent a novel pharmacological target in liver cirrhosis.

Although the β3-AR agonists, mirabegron and vibegron, have been approved for the treatment of overactive bladder [71], their use in other conditions, such as obesity or metabolic diseases, has not yet been approved, and research in these areas is currently under investigation [70,71,73]. However, the increasing use of the β3-agonist mirabegron in the treatment of overactive bladder has highlighted the need for clinicians to be aware of unexpected adverse effects that are not documented in the drug’s posology or identified in clinical trials [74,89,90]. A recent pharmacovigilance analysis of the FDA Adverse Event Reporting System (FAERS) database revealed new significant adverse reactions associated with this type of β3-agonist, including arrhythmia, palpitations, dementia, transient ischemic attack, Parkinson’s disease, antineutrophil cytoplasmic antibody (ANCA)-positive vasculitis, and allergic reactions such as angioedema [90]. These aspects underscore the necessity of large-scale clinical trials to validate the long-term efficacy and safety of β3-agonists.

## 4. Conclusions and Future Perspectives

This review highlights the increasing importance of beta-adrenergic modulation in managing liver cirrhosis, broadening its therapeutic scope from stabilising blood flow to influencing fibrogenic and inflammatory processes. Growing evidence points to adrenergic signalling—especially through hepatic stellate cells and immune-metabolic pathways—in the development and progression of fibrosis. In this context, β3-adrenergic receptors emerge as promising targets, supported by experimental research showing their antifibrotic and immunomodulatory effects.

Future directions should focus on translational strategies that integrate molecular characterization, non-invasive biomarker assessment, and receptor-specific pharmacological profiling. The development of targeted delivery platforms, including nanocarrier-based systems, may optimize tissue selectivity and enhance therapeutic efficacy. Clinical implementation of such approaches will require prospective studies evaluating intrahepatic and systemic outcomes, including fibrosis dynamics and biomarker trajectories. Ultimately, refining adrenergic interventions based on mechanistic insights may support a more personalized approach to cirrhosis management, particularly in the compensated stages of the disease.

## Figures and Tables

**Table 1:** Studies that show the superiority of carvedilol in compensated cirrhosis.

Type of Study	Number of Patients	Main Findings	Limitations	Reference
RCT	Carvedilol-14 Propranolol-14 Placebo-7	HVPG was lowered more after carvedilol (decreased by 20%) than after propranolol (decreased by 13%) at 1 h HVPG was lowered consistently more with carvedilol (64%) than with propranolol (14%)	Low number of patients included, short-term follow-up, single center design, limited patient heterogeneity, primary focus on surrogate outcomes, not direct clinical endpoints	[29]
RCT, prospective	Carvedilol-26 Propranolol-25	HVPG was lowered more after carvedilol (decreased by 19%) than after propranolol (decreased by 12%) at 1 h HVPG was lowered consistently more with carvedilol (54%) than with propranolol (23%)	Low number of patients included, short-term follow-up, single center design, limited patient heterogeneity, primary focus on surrogate outcomes, not direct clinical endpoints	[30]
Prospective, non-randomised	Carvedilol-10	HVPG was lowered by ~23% with carvedilol after 1 h	Low number of patients included, lack of control group, short-term follow-up, primary focus on surrogate outcomes, not direct clinical endpoints, potential for measurement bias	[31]
Prospective, non-randomised	Carvedilol-38 Propranolol-37 EVL-29	Carvedilol has better lowering of HVPG effects than propranolol + carvedilol has a hemodynamic response in Propranolol non-responders	Lack of randomization, restriction to compensated cirrhotic patients, empirically defined dosing categories, absence of blinding, no formal dose-finding for carvedilol, potential underestimation of side effects, and limited generalizability to routine practice.	[32]
Systematic review with metanalyses	5 RCT-175 patients	Carvedilol lowers portal hypertension more than propranolol, but without an accurate comparison of the adverse effects	Low number of patients and trials included, heterogeneity in study design, poor trial quality, inadequate adverse event reporting	[33]
RCT, prospective	Carvedilol-30 patients Propranolol-29 patients	Carvedilol showed a better HVPG response 1 month after variceal bleeding (75%) than propranolol (50%)	High risk of bias (study not double-blinded), short-term follow-up, primary focus on surrogate outcomes, not direct clinical endpoints, small sample size, selection bias	[28]
Systematic review of multiple RCTs	10 RCT-810 patients	Carvedilol is superior to other beta-blockers in lowering HVPG	Low heterogeneity of included studies, small sample sizes, short follow-up periods, high risk of bias, insufficient power	[25]
Metanalyses	4 RCT-352 patients (Carvedilol-181, Control-171)	Survival was improved, and a lowered risk of decompensation was achieved, primarily in the form of ascites, with carvedilol therapy	Partial blinding, endpoint variability, risk of publication/data availability bias, limited applicability to decompensated cirrhosis, small sample sizes in some subgroups, and potential for bias in open-label designs.	[27]

HVPG, hepatic venous pressure gradient; NSBB, non-selective beta-blocker; CSPH, clinically significant portal hypertension; RCT, randomised controlled trial; EVL, endoscopic variceal ligation.

**Table 2:** Biomarkers of non-invasive monitoring of NSBB efficacy in cirrhosis.

Type of Molecule	Main Findings	Type of Study	Clinical Applicability/Limitations	References
sVCAM-1	Serum levels are significantly higher in decompensated cirrhosis (*p* < 0.01) moderate correlation between the variation in HVPG and sVCAM-1 after treatment with NSBBs (*p* = 0.03) Elevated levels in decompensated cirrhosis; correlated with Child–Pugh, MELD, and hyperdynamic circulation. Associated with sVAP-1 in early disease stages; currently under evaluation in NSBB treatment response (NCT03720067).	Retrospective and prospective cohort studies; ongoing interventional trial	No standardized cut-off values; not yet validated for clinical use; under investigation in clinical trials	[43,58,59]
IL-6, IL-10, IFN-γ	Higher levels of IL-6, IL-10, IFN- γ—more clinical events; NSBBs treatment—significantly better event-free survival, including hepatocellular carcinoma, death, and the need for liver transplantation (HR = 0.36, 95%CI = 0.18–0.71) NSBB therapy modulates immune response and improves survival. IL-6 > 37 pg/mL predicts short-term complications.	Prospective longitudinal and case-control studies	Promising prognostic value, particularly for IL-6; clinical implementation needs further validation	[43,45,59,60]
ET-1	Peripheral ET-1 levels significantly increased after NSBB treatment (from 1.33 fmol/L to 3.0 fmol/L—*p* = 0.032) Serum ET-1 levels are significantly increased in cirrhotic patients and correlate with HVPG and liver dysfunction. NSBB therapy is associated with increased ET-1 levels in non-responders, suggesting a role in intrahepatic vasoconstriction and therapeutic resistance. A related cytokine imbalance involving TGF-β1 may further influence portal pressure and variceal development.	Prospective observational studies	Potential indicator of hepatic vascular tone; clinical utility remains exploratory and requires validation in interventional studies	[53,61]
TGF-β2	Peripheral levels of TGF-b2 significantly decreased and maintained their low levels, compared to the controls (from 263.17 pg/mL to 180.18 pg/mL, *p* < 0.001); lower levels of peripheral TGF- β2 in normalised HVPG patients in comparison to the patients without HVPG normalisation (*p* = 0.033) Peripheral levels of TGF-β2 decrease significantly in patients treated with NSBBs and remain low over time. Lower TGF-β2 is associated with HVPG normalization, suggesting a link to vascular remodeling and response to therapy.	Prospective observational study	potential marker of endothelial and fibrotic modulation under NSBBs; clinical use is exploratory and requires further validation.	[53,62]
vWF	Increased levels of vWF were correlated with disease severity (*p* < 0.001); increased levels of vWF were predictive of decompensation/liver-related mortality (*p* = 0.031); Elevated vWF levels are significantly associated with advanced liver disease severity, increased risk of hepatic decompensation or liver-related mortality, and correlate with the degree of portal hypertension and reduced transplant-free survival. Decreases in vWF levels after NSBB therapy were linked to better outcomes.	Retrospective analysis of prospectively characterized cohort	Promising non-invasive marker of portal hypertension severity; lacks standardization for routine prognostic use.	[55,63]
miR-181b-5p	Levels at 1 year, but not at baseline, for patients who achieved HVPG normalization were higher in patients who developed ascites efficient in predicting decompensation (ascites) AUC = 0.7 (95% CI 0.59–0.78), *p* < 0.01 Predicts ascites onset in compensated cirrhosis and is associated with decompensation risk (AUC = 0.7). May support early risk stratification.	Prospective studies	Not yet validated for NSBB response monitoring. Prognostic role under investigation.	[56,57,64]

sVCAM-1—soluble vascular cell adhesion molecule-1, IL-6—interleukin 6, IL-10—interleukin 10, IFN-γ—interferon-gamma, ET-1—endothelin-1, TGF-β2—transforming growth factor-beta 2, vWF—von Willebrand Factor, miR-181b-5p—MicronRNA-181b-5p.

**Table 3:** Adrenergic modulation in portal hypertension: comparative profile of β1 and β2 blockers and β3 agonists.

	β1 Blockers	β2 Blockers	β3 Agonists
Primary site of pharmacological action	Myocardium	Splanchnic vasculature	Endothelial cells, liver, adipose tissue
Mechanism of action and receptor specificity	Decrease heart rate and cardiac output → ↓ portal inflow [20,21,22]	Induce splanchnic vasoconstriction → ↓ portal venous flow [22,23,72]	Stimulate NO production and vasodilation via endothelial β3-AR activation [73,74,75,76]
Hemodynamic effect on (portal circulation)	Reduce portal pressure via decreased cardiac output [23,28]	Reduce portal inflow via vasoconstriction of splanchnic circulation [21,22,23]	Lower intrahepatic vascular resistance and portal pressure [73,76]
Effect on systemic arterial pressure	Moderate reduction; hypotension risk at high doses [22,30]	Mild systemic effect; usually used with β1 blockers [23,72]	Minimal systemic impact: preserves mean arterial pressure [73,76,77]
Common side effects	Bradycardia, hypotension, fatigue [7,22,23]; β1-selective blockers rarely cause bronchospasm, but caution is advised in asthma or COPD, especially at high doses.	Bronchospasm, systemic hypotension; caution in cirrhosis and respiratory disease [23]	Favorable tolerability profile; minimal cardiovascular effects; safety under ongoing investigation [73,78,79,80]
Therapeutic limitations due to safety profile	Heterogeneous response; adverse events; genetic polymorphisms (β-AR) may affect efficacy [10,21,25]	Not used as monotherapy due to limited tolerability [21]	Not currently approved for portal hypertension; additional clinical evidence is required [73,74,75]
Clinical evidence and use in cirrhosis	Standard of care in cirrhosis (e.g., propranolol, carvedilol); proven efficacy [10,23,28,29,30]	Used as component of NSBBs; enhances β1 effect; no role alone [23,72]	Preclinical and early clinical studies indicate potential efficacy; not yet integrated into standard practice [64,65,67,70,73]
Pleiotropic effects and potential therapeutic benefits	Reduces risk of variceal bleeding, decompensation; potential antifibrotic effect [10,29]	Enhances β1 blockade in NSBBs; synergistic in reducing portal pressure [23]	Antifibrotic, anti-inflammatory, improves metabolic profile; induces NO-mediated vasodilation [73,74,75,76,77,78,79,80]

NO—nitric oxide, COPD—chronic obstructive pulmonary disease, β-AR—beta receptor, NSBB—non-selective beta-blocker.

## Data Availability

No new data were created or analysed in this study.
